# Intraoperative quantification of floating mass transducer coupling quality in active middle ear implants: a multicenter study

**DOI:** 10.1007/s00405-020-06313-z

**Published:** 2020-09-03

**Authors:** Laura Fröhlich, Torsten Rahne, Stefan K. Plontke, Tobias Oberhoffner, Rüdiger Dahl, Robert Mlynski, Oliver Dziemba, Aristotelis Aristeidou, Maria Gadyuchko, Sven Koscielny, Sebastian Hoth, Miriam H. Kropp, Parwis Mir-Salim, Alexander Müller

**Affiliations:** 1 Department of Otorhinolaryngology, Head and Neck Surgery, Martin Luther University Halle-Wittenberg, University Medicine Halle (Saale), Ernst-Grube-Str. 40, 06120 Halle (Saale), Germany; 2 Department of Otorhinolaryngology, Head and Neck Surgery “Otto Körner”, Rostock University Medical Center, Rostock, Germany; 3 Department of Otorhinolaryngology, Head and Neck Surgery, University Medicine of Greifswald, Greifswald, Germany; 4 Department of Otorhinolaryngology, Institute of Phoniatry/Pedaudiology, Jena University Hospital, Friedrich-Schiller-University Jena, Jena, Germany; 5 Present Address: Department of Otorhinolaryngology, Helios Clinic, Erfurt, Germany; 6 Department of ENT, University of Heidelberg, Heidelberg, Germany; 7 ORL Department Friedrichshain Clinic, Vivantes Hearing Center, Berlin, Germany

**Keywords:** Coupling quality, Active middle ear implant, Floating mass transducer, Objective measures, Intraoperative

## Abstract

**Purpose:**

Evaluating the effectiveness of intraoperative auditory brainstem responses (ABRs) to stimulation by the Vibrant Soundbridge (VSB) active middle ear implant for quantifying the implant’s floating mass transducer (FMT) coupling quality.

**Methods:**

In a diagnostic multicentric study, patients (> 18 years) who received a VSB with different coupling modalities were included. Pre- and postoperative bone conduction thresholds, intraoperative VSB-evoked ABR thresholds (VSB-ABR) using a modified audio processor programmed to preoperative bone conduction thresholds, postoperative vibrogram thresholds, and postoperative VSB-ABR thresholds were measured. Coupling quality was calculated from the difference between the pure tone average at 1000, 2000, and 4000 Hz (3PTA) vibrogram and postoperative 3PTA bone conduction thresholds.

**Results:**

Twenty-three patients (13 males, 10 females, mean age 56.6 (± 12.5) years) were included in the study. Intraoperative VSB-ABR response thresholds could be obtained in all except one patient where the threshold was > 30 dB nHL. Postoperatively, an insufficient coupling of 36.7 dB was confirmed in this patient. In a Bland–Altman analysis of the intraoperative VSB-ABRs and coupling quality, the limits of agreement exceeded ± 10 dB, i.e., the maximum allowed difference considered as not clinically important but the variation was within the general precision of auditory brainstem responses to predict behavioral thresholds. Five outliers were identified. In two patients, the postoperative VSB-ABR thresholds were in agreement with the coupling quality, indicating a change of coupling before the postoperative testing.

**Conclusion:**

The response thresholds recorded in this set-up have the potential to predict the VSB coupling quality and optimize postoperative audiological results.

## Introduction

Active middle ear implants (AMEIs) have become an appropriate solution for hearing rehabilitation in patients with moderate to severe sensorineural or mixed hearing loss who cannot use conventional hearing aids due to technical issues such as feedback or sound distortion, or patient-related issues like recurrent infections of the auditory canal [1]. The Vibrant Soundbridge (VSB) (MED-EL, Innsbruck, Austria) is a frequently used AMEI which transforms airborne sound into mechanical vibrations by its miniature floating mass transducer (FMT) [2, 3]. When the VSB was originally designed for treatment of sensorineural hearing loss, the only option to couple the FMT to the middle ear was to crimp it onto the long process of the incus [4]. The indication criteria have been considerably extended, now also including patients with conductive or mixed hearing loss, since the direct drive stimulation overcomes the patient’s conductive hearing loss and provides a more efficient sound transfer than can be achieved with conventional hearing aids [5, 6]. The development of different coupling modalities has led to a large variety of surgical applications referred to as “vibroplasty”. The FMT can be coupled to the long process (LP) of the incus [4], the short process (SP) of the incus [7], the stapes suprastructure, the round window (RW) membrane [8], or the oval window (OW) [9, 10], i.e., the stapes footplate.

For postoperative hearing threshold and speech recognition improvement, a sufficient energy transfer from the FMT to the inner ear is essential. The energy transfer is determined by the coupling quality of the FMT. “Vibroplasty in situ thresholds”, also referred to as “vibrogram” (VIB) thresholds, can be measured as behavioral thresholds by ordinary pure-tone audiometry with stimulation via the implanted FMT and compared to the bone conduction (BC) thresholds. The coupling quality can be calculated for each frequency or as an average at certain audiometric test frequencies: Coupling quality (dB) = VIB – BC.

A deterioration of coupling quality is indicated by increasing VIB thresholds, i.e., increasing difference between VIB and BC thresholds. The loss of energy transfer has to be overcome by the system by additional amplification. In patients who just meet the inclusion criteria of the VSB, poor coupling quality can, therefore, result in limited dynamic range and insufficient audiological outcome with the AMEI [6]. The VIB thresholds are widely used as an outcome measure of treatment with the VSB [7, 11–14]. Müller et al. reported that good word recognition in VSB patients could less likely be achieved, if the coupling quality was reduced by more than 20 dB [15].

Revision surgery can become necessary when the coupling deteriorates over time or directly after surgery when the coupling quality is found to be insufficient in the vibrogram measurement. The long-term data on revision rates due to insufficient coupling vary between 3.4% [16], 8.7% [17], 9.5% [18], and up to 15.6% [19].

During vibroplasty surgery, the surgeon can only rely on subjective, tactile judgement, while objective and quantitative feedback of the coupling quality is highly desirable but not available yet. To provide such feedback, the VIB thresholds have to be measured objectively. It has been shown that the recording of auditory evoked potentials (AEPs), like auditory steady state responses (ASSRs) [20], compound action potentials (CAPs) [21–23], and auditory brainstem responses (ABR) [24–26] is possible in AMEI patients. Custom made set-ups were used in these studies for providing stimulation by the implanted transducer. However, most of the studies lack a sufficient sound- or vibration-level calibration of the set-up allowing only a relative assessment, i.e., comparing one FMT coupling position to another. However, Fröhlich et al. [26] have shown that measuring VSB-evoked ABRs using the experimental set-up already described by Radeloff et al. [21] was feasible to quantify the FMT coupling quality in a VSB patient undergoing revision surgery.

The aim of this study was to compare the intraoperative VSB-evoked ABR thresholds to the postoperative coupling quality as the reference standard to evaluate the agreement between the two methods. The feasibility of the VSB-evoked ABR to predict the FMT coupling quality was evaluated in a series of patients with different coupling modalities in multiple implant centers.

## Materials and methods

### Study participants

Participants were patients older than 18 years, who were regularly scheduled for hearing rehabilitation with the active middle ear implant or for revision surgery, thus meeting the audiological and clinical criteria of the manufacturer (absence of active middle ear infections; ability to get benefit from amplification; ear anatomy allows FMT positioning; stable BC thresholds ≤ 45 dB HL at 500 Hz, ≤ 50 dB HL at 1000 Hz, ≤ 55 dB at 1500 Hz, and ≤ 65 at 2000, 3000, and 4000 Hz). Patients suffering from retro-cochlear, or central auditory disorders as well as patients suffering from conditions that would interfere with the ability to adequately perform the psychoacoustic tests were excluded from the study. If postoperative BC thresholds deteriorated by more than 10 dB compared to preoperative BC thresholds, the patients were excluded from the study as well.

The study was designed as a prospective multicenter clinical study at five tertiary referral centers. Written informed consent was obtained from all patients before enrollment to this study. The study protocol was reviewed and approved by the local ethics committee at the institution of the principal investigator (approval number: 2018–34).

### Experimental set-up

The Eclipse EP25 (Interacoustics A/S, Middelfart, Denmark) was used for stimulation and recording. ER-3A insert earphones (3 M, St. Paul, MS, USA) were connected to the headphone output as for ordinary AEP measurements. Signal transmission to the implant was provided by connecting the sound tube of the insert earphone to a type 404 audio processor (AP404) (MED-EL, Innsbruck, Austria) by means of a sound tube adapter glued to the audio processor’s single microphone aperture. The gain of the audio processor was set according to the patients’ preoperative BC thresholds. The output limitation, compression, and special options (noise reduction, speech enhancement features, etc.) were deactivated. Broadband CE-Chirps presented with a rate of 49.1 Hz and alternating polarity were used for stimulation. ABRs were recorded in a two-channel set-up using self-adhesive surface electrodes. The skin was prepared to provide impedances of 5 kΩ or less. The electrodes were placed at the hairline (active), approximately 1 cm below this electrode (ground), and on the mastoids (reference). For intraoperative recordings, the ipsilateral electrode was placed at the neck to provide adequate distance to the surgical field. The EEG signal was sampled at 30 kHz with an A/D resolution of 16 bits. A bandpass filter of 33–1500 Hz was applied to the EEG signal. The artifact rejection level was set to 40 μV. Responses were averaged to at least 1000 stimuli for intraoperative and 2000 stimuli for postoperative recordings. If the residual noise was 40 nV or less for intraoperative and 80 nV or less for postoperative measurements, the recording was stopped earlier.

The calibration of the set-up was based on the use of calibrated stimuli and transducers (insert earphones) from the stimulation and recording system and the audio processor setting to the BC thresholds compensating for the patient’s hearing loss. Stimuli of short duration (such as chirps) are normally calibrated according to “dB nHL” as the sound pressure level in an acoustic coupler (of specified 2 cm^3^ cavity and shape), (ISO 389–6). The sound tube of the insert earphone coupled to the microphone aperture of the audio processor and the audio processor driving the implanted FMT does not correspond to the acoustic coupler so that the exact level at the FMT is unknown. However, all thresholds measured in the described experimental set-up will be referred to as in “dB nHL” according to the lowest ABR stimulus intensity at which a VSB-evoked ABR can be detected.

### Technical investigation of the set-up

The input–output function and signal transmission of the experimental set-up were measured to investigate the set-up’s technical limitations with respect to interpretation of the study results.

For recording of the input–output function, the AP404 was placed over a demodulator (AP-adapter, MED-EL, Innsbruck, Austria). ER-3A insert earphones were connected to the Eclipse and the insert earphone sound tube was connected to the AP404 microphone aperture. The set-up was the same as described before. The output from the demodulator was recorded for ABR stimulus intensities (input) between 0 and 100 dB nHL using an Infinii Vision 2000 X-Series oscilloscope (Keysight Technologies, Santa Rosa, CL, USA). The measurement was repeated for arbitrary settings of the AP404 to 0, 20, and 40 dB HL BC thresholds.

For measurement of the frequency-dependent VSB signal transmission of the complete signal chain (ER-3A insert earphones with sound tube, AP404, FMT), the AP404 was placed over an experimental implant, a modified VORP502 (MED-EL, Innsbruck, Austria) from which the FMT was cut and replaced by connection cables. The AP404 was programmed to 0 dB HL BC threshold. The ABR stimulation in the Eclipse was set to bilateral to record the original signal from the left headphone output and to use the right headphone output for recording the signal at the FMT after stimulation with the complete signal chain. The original ABR input signal and the signal at the FMT were recorded for tone bursts of 500, 1000, 2000, and 4000 Hz as well as the broadband CE-Chirp.

### Procedures

#### Preoperative

Before surgery, the patient’s air conduction (AC) and BC thresholds (preoperative BC and AC) were measured as behavioral pure-tone thresholds. At each study center, the clinical routine audiometers and transducers (circumaural headphones and BC transducers) were used. All measurements were performed with calibrated instruments in a soundproof room [28].

#### Intraoperative

During surgery, the VSB-evoked ABRs (intraoperative VSB-ABR) were recorded using the experimental set-up described before. The recording electrodes were positioned before draping of the surgical field. The ABRs were recorded after positioning the FMT and during or shortly after wound closure. To reduce muscle artifacts especially from the neck muscles, the anesthesiologist was instructed to keep the patient’s level of anesthesia constant as during the rest of the surgery. The measurements were started at a stimulus intensity of 30 dB nHL and wave V was identified. The stimulus intensity was then decreased in steps of 10 dB and increased in steps of 5 dB until the threshold was reached (ascending descending method). At threshold level, the recording was repeated for reproducibility.

#### Postoperative

Six weeks after surgery, the patient’s BC pure tone thresholds (postoperative BC) were measured.

Prior to the initial fitting of the patient’s audio processor, the VIB thresholds were measured as behavioral thresholds in the Symfit fitting software (MED-EL, Innsbruck, Austria) within Connexx software (Sivantos GmbH under Trademark License of Siemens AG, Erlangen, Germany). To reduce the risk of masking by inherent noise of the audio processor, a Samba Lo audio processor (MED-EL, Innsbruck, Austria) was used.

The VSB-ABR measurement was repeated postoperatively (postoperative VSB-ABR) using the same experimental set-up and procedure as intraoperatively including the setting of the audio processor according to the preoperative BC thresholds.

#### Data analysis

Descriptive statistics were used to report demographic (e.g., age and gender) and baseline characteristics (e.g., clinical conditions and previous otosurgery). Quantitative data were presented as mean, standard deviation, and range (minimum and maximum), qualitative data were presented as graphs if appropriate. Graphs were created in GraphPad Prism 8 (Graphpad Software, San Diego, CA, USA). Hearing thresholds exceeding the measurement limit of the audiometers (unmeasurable thresholds) were set to 110 dB HL for the calculation of mean hearing thresholds.

The coupling quality was calculated from the difference between the VIB and postoperative BC thresholds as a frequency specific measure and as pure tone average (PTA). The coupling qualities were then compared to the intraoperative VSB-ABR thresholds by a Bland–Altman analysis. The differences between VIB and BC thresholds (the coupling quality) as the gold standard and the intraoperative VSB-ABR thresholds, were plotted against the averages of these two measures. Horizontal lines were drawn at the mean difference and the 95% limits of agreement defined as the mean difference ± 1.96 times the standard deviation of the differences. Ninety-five percent of the differences are expected to lie within these limits of agreement. The maximum allowed clinically relevant difference between the two techniques was set to 10 dB. If the measurement error between the two methods exceeded our tolerance level (i.e., ± 10 dB) in more than 5% of the study population, the two methods could not be considered equivalent. Limits of agreement not exceeding the maximum allowed difference were considered to be not clinically important so that the two methods would be considered to be in good agreement.

The VSB-ABR method was further analyzed for systematic bias, proportional error, and dependence of the method’s variation on the magnitude of measurements. To detect statistically significant systematic bias, the mean of the difference was compared to 0 (no difference between the two methods) by a one-sample *t* test. Statistical significance was set to *p* < 0.05. SPSS 25 for Windows software (IBM, Armonk, NY, USA) was used for all statistical analyses.

Outliers in the Bland–Altman plot were identified as data points where the difference exceeded the ± 10 dB maximum allowed difference. For outliers, the postoperative VSB-ABR threshold was compared to the coupling quality. Thus, possible changes of coupling quality from the time of the intraoperative VSB-ABR measurement to the time of the VIB measurement (on which the calculation of coupling quality was based) were sought to be identified as a possible cause for discrepancy.

## Results

### Technical investigation of the set-up

**Fig. 1:**
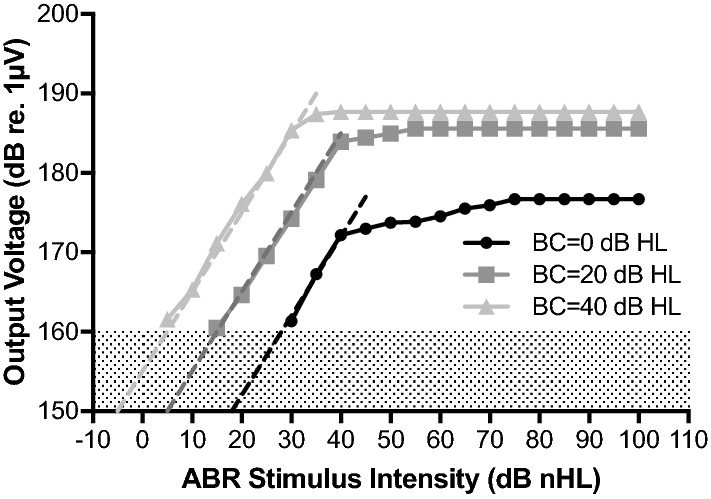
Output level of the AP404 with the insert earphone sound tube attached to its microphone for increasing ABR stimulus intensities. The input was the broadband CE-Chirp. The AP404 was programmed to arbitrary BC thresholds of 0, 20, and 40 dB HL. The dashed lines illustrate linear increase with a slope of 1 dB re. 1 µV/1 dB nHL. The dotted area represents the noise floor

The input–output function of the experimental set-up is illustrated in Fig. 1. Due to signal noise, the output could not be recorded for ABR stimulus intensities lower than 30 dB nHL for 0 dB HL BC setting and lower than 15 dB nHL for 20 dB HL BC setting of the AP404. The output level increased linearly with a slope of 1 dB re. 1 µV/1 dB nHL. The output level saturated at 35 dB nHL stimulus intensity for 40 dB HL BC setting and at 40 dB nHL for 20 and 0 dB HL BC setting. For programming of the AP404 to 0 dB HL BC thresholds, the ABR stimulus intensity at which the saturation occurred was not as clear. The output saturation level, i.e., the maximum output, for the 0 dB HL BC setting was approximately 15 dB lower than for the 20 and 40 dB HL BC settings.

**Fig. 2:**
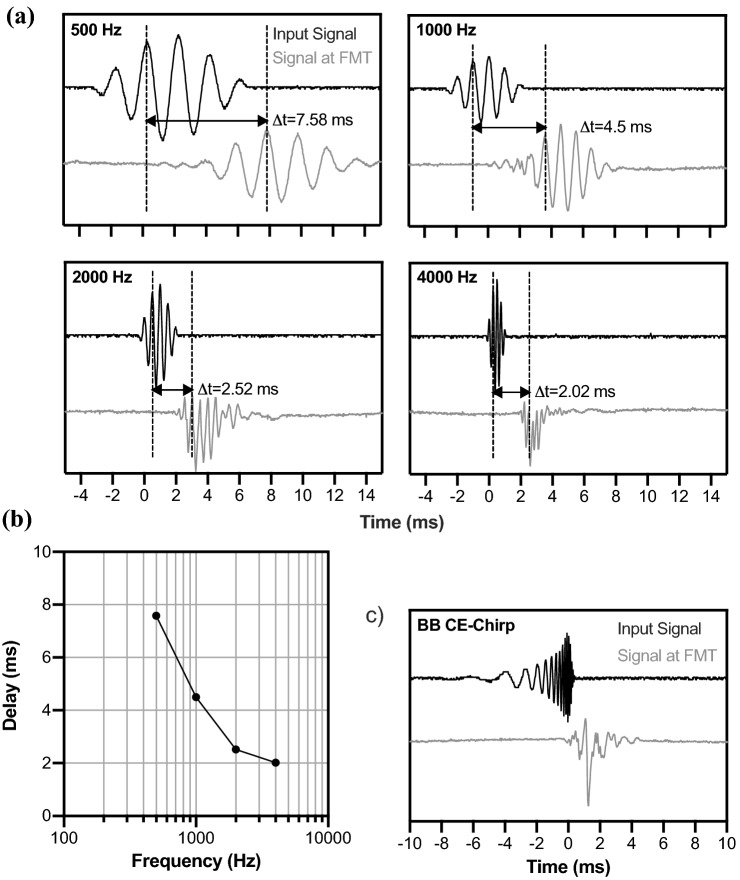
Stimulus transmission of the AP404 with the insert earphone sound tube attached to its microphone. **a** Temporal waveforms of 500, 1000, 2000, and 4000 Hz tone bursts. The back line shows the original signal, the gray line shows the signal at the FMT. **b** Frequency-specific delay time function for transmitted tone bursts. **c** Transmission of the broadband CE-Chirp emerging as a click-like stimulus at the FMT

The signal transmission of the AP404 with an attached insert earphone sound tube showed frequency-specific delay. For 500 Hz tone bursts, the delay was 7.58 ms; for 4000 Hz, it was 2.02 ms (see Fig. 2a). The transmitted signals were burst-like except for the 4000 Hz tone burst. The frequency specific delay is illustrated in Fig. 2b. The output for the broadband CE-Chirp, i.e., the stimulus used in the study, is illustrated in Fig. 2c. With the frequency-specific delay, the broadband CE-Chirp was transmitted as a click-like stimulus at the FMT.

### Clinical results

In the course of the clinical study, intraoperative measurements were performed in 30 patients. Seven patients had to be excluded from the study, six due to postoperative deterioration of the BC thresholds by more than 10 dB, and one due to a calcified round window as was observed intraoperatively. The data sets of 23 patients were, therefore, included in the study (see Table 1).

**Table 1:** Characterization of study participants

ID	Age (years)	Gender	Implanted side	Reason for implantation	Vibroplasty	Coupler	Preop. 4PTA (dB HL)	
							BC	AC
1	50	M	L	Multiple ME surgeries, CWD, ME fibrosis	RW vibroplasty	RW soft	23.75	58.75
2	56	F	R	Multiple canaloplasties, stenosis of external auditory canal, recurrent OE	PORP vibroplasty	CliP	2.00	51.25
3	51	M	L	Multiple ME surgeries, ME fibrosis	OW vibroplasty	No coupler	27.50	56.25
4	53	M	L	Multiple ME surgeries, initial stapes vibroplasty, revision with RW vibroplasty, FMT dislocation, revision	PORP vibroplasty	CliP	43.75	60.00
5	60	M	L	Previous ME surgery, ME fibrosis, recurrent OE and myringitis with HA	Stapes vibroplasty	SMPX on stapes	43.75	76.25
6	39	F	R	Multiple ME surgeries, recurrent cholesteatoma	TORP vibroplasty	OW	33.75	45.00
7	39	F	L	Chronic OE	SP incus vibroplasty	SP	42.50	51.25
8	51	F	L	Multiple ME surgeries, recurrent OE, initial stapes vibroplasty, revision	RW vibroplasty	No coupler (cartilage)	43.75	98.75
9	34	F	R	Multiple canaloplasties, fixation of stapes footplate, ME fibrosis	SP Incus vibroplasty	SP	18.75	73.75
10	33	F	R	VORP implant migration with FMT dislocation, revision	RW vibroplasty	RW	37.50	76.25
11	72	M	L	Multiple ME surgeries, CWD, ME fibrosis	PORP vibroplasty	CliP	25.00	65.00
12	67	M	L	Multiple ME surgeries, ME fibrosis, arrosion of stapes footplate and PL fistula OW	RW vibroplasty	No coupler	47.50	> 85.00
13	59	M	L	Multiple canaloplasties, stenosis of external auditory canal	SP incus vibroplasty	SP	41.25	53.75
14	58	F	R	Re-implantation after VORP implant protrusion through skin	TORP vibroplasty	OW	38.75	57.50
15	60	M	L	Recurrent cholesteatoma, multiple FMT repositioning, FMT dislocation, revision	TORP vibroplasty	OW	43.75	81.25
16	81	M	L	Multiple ME surgeries, chronic OM	PORP vibroplasty	CliP	40.00	80.00
17	52	F	L	Multiple ME surgeries, ME fibrosis, atelectasis	PORP vibroplasty	CliP	12.50	57.50
18	67	M	L	Multiple ME surgeries, cholesteatoma, ME fibrosis	TORP vibroplasty	OW	42.50	82.50
19	67	M	L	Stenosis of external auditory canal, chronic OM and OE	TORP vibroplasty	OW	33.75	55.00
20	59	F	R	SNHL, unable to use HA due to hyperhidrosis	LP Incus vibroplasty	LP	43.75	46.25
21	77	M	R	Multiple ME surgeries, recurrent cholesteatoma	OW vibroplasty	RW soft	40.00	101.25
22	55	M	R	Microtia	PORP vibroplasty	CliP	37.50	90.00
23	60	F	L	Multiple ME surgeries, lateral petrosectomy, PL fistula OW	RW vibroplasty	RW	51.25	> 102.50

*CWD* Canal wall down, *HA* hearing aid, *LP* long process, *ME* middle ear, *OM* otitis media, *OE* otitis externa, *OW* oval window, *PL* perilymph, *PORP* partial ossicular replacement prosthesis, *RW* round window, *SMPX* Symphonix Coupler, *SP* short process, *SNHL* sensorineural hearing loss, *TORP* total ossicular replacement prosthesis

Patients were between 33 and 81 years old with a mean age of 56.6 (± 12.5) years. Thirteen patients were male, ten were female. The left ear was implanted in 15 patients and the right ear was implanted in eight patients. The mean 4PTA BC thresholds of the participants was 36.4 (± 9.9) dB HL, the mean 4PTA AC threshold was 69.8 (± 18.0) dB HL. The AC hearing thresholds exceeded the measurement limit of the audiometers in two patients. Thresholds at the affected frequencies were set to 110 dB for the calculation of the mean 4PTA AC threshold. Six patients received a CliP-, three a SP-, one a LP-, two a RW-, two a RW-soft- (one of those on the stapes footplate), five an OW-, and one a Symphonix-Coupler modified for placement on the stapes suprastructure. In three patients, no specific coupler was used and the FMT was placed either directly on to the RW (*n* = 2) or with a small piece of cartilage between the FMT and the RW (*n* = 1).

The intraoperative VSB-ABR was measured in all patients. A response threshold was obtained in all but one patient (#6) where no potentials could be recorded at all. Based on the results of the input–output function, the threshold was marked as > 30 dB nHL in this patient. The response thresholds of the other 22 patients were between 0 and 20 dB nHL with a mean intraoperative VSB-ABR threshold of 9.8 (± 6.7) dB nHL. The postoperative VSB-ABR was measured in 19 patients. Response thresholds were obtained in 16 patients. In three patients, artifacts interfered with the responses so that thresholds could not be obtained.

**Fig. 3:**
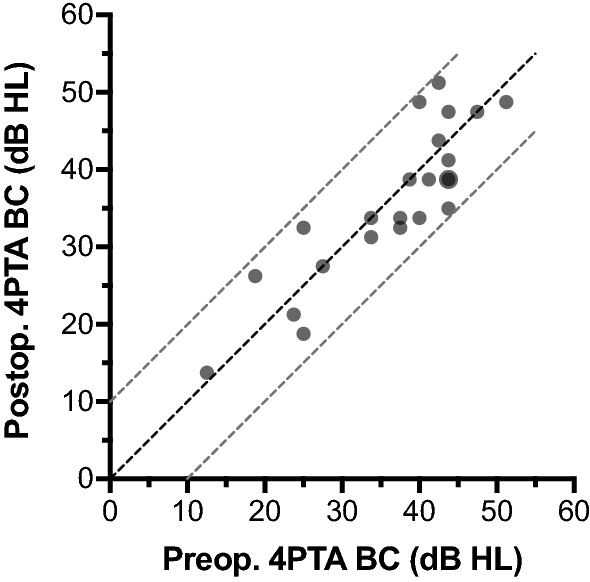
Comparison of pre- and postoperative BC thresholds. Overlapping data points are displayed larger. The diagonal lines are the lines of equal thresholds as well as the ± 10 dB limits

**Fig. 4:**
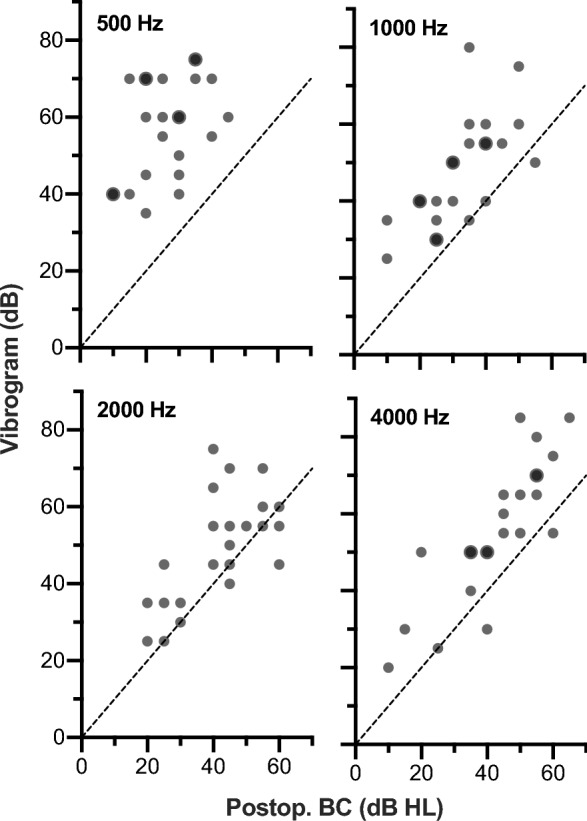
Frequency-specific coupling qualities of the included patients as shown by the VIB thresholds plotted against the postoperative BC thresholds. Overlapping data points are displayed larger. The diagonal lines are the lines of equal thresholds, i.e., perfect coupling quality

Postoperatively, the 4PTA BC threshold was 35.8 (± 10.0) dB HL (postoperative BC). The pre- and postoperative BC thresholds of all included patients are shown in Fig. 3. The VIB thresholds could be measured in all 23 patients and the mean was 52.4 (± 12.6) dB, so that the mean 4PTA coupling quality was 16.6 (± 8.7) dB. The coupling quality of patient #6, where no intraoperative VSB-ABR threshold could be measured (marked as > 30 dB nHL), was 36.7 dB nHL. The mean frequency specific coupling quality was 30.9 (± 12.4) dB at 500 Hz, 15.0 (± 10.7) dB at 1000 Hz, 7.6 (± 11.4) dB at 2000 Hz, and 12.8 (± 10.1) dB at 4000 Hz. The frequency-specific coupling quality for all individual patients is illustrated in scatter plots in Fig. 4 showing the VIB thresholds against the postoperative BC thresholds. The illustration as well as the mean coupling quality and standard deviation show that the largest variation of coupling quality was at 500 Hz.

Based on the results of the technical examination showing a significant delay of signal transmission especially at 500 Hz as well as the broad distribution of coupling quality at 500 Hz, the pure tone average at 1000, 2000, and 4000 Hz was used for all further analyses (3PTA).

**Fig. 5:**
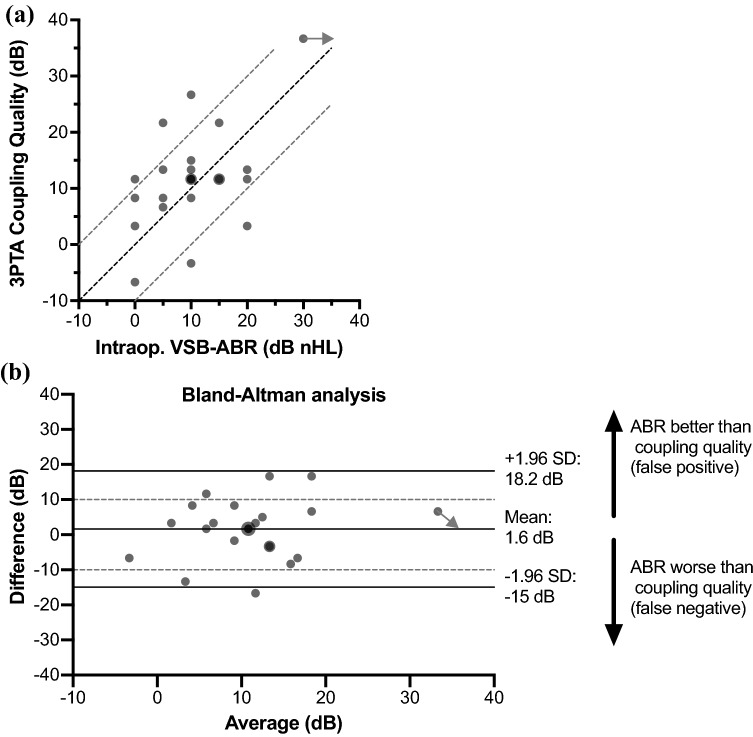
Analysis of the intraoperative VSB-ABR thresholds in relation to the 3PTA coupling qualities of all patients. Overlapping data points are displayed larger. For the patient where the intraoperative VSB-ABR measurement did not show a response, the data point is marked by an arrow (assumed threshold > 30 dB nHL). **a** Scatterplot of 3PTA coupling qualities and intraoperative VSB-ABR thresholds. The diagonal lines are the line of equal thresholds as well as the ± 10 dB limits. **b** Bland–Altman analysis plotting the differences between VIB and BC thresholds (3PTA coupling qualities) and the intraoperative VSB-ABR thresholds against the averages of these two measures. The solid horizontal lines show the mean of the differences and the limits of agreement (mean difference ± 1.96 standard deviations). The dotted lines show the ± 10 dB which we allowed as a maximum acceptable (i.e., clinically not relevant) difference between the two methods. Limits of agreement exceeding our tolerance level (± 10 dB) indicated disagreement between the two measurements

The 3PTA coupling qualities are plotted in relation to the intraoperative VSB-ABR thresholds for all patients in a scatterplot in Fig. 5a. The distribution of coupling qualities was narrow, i.e., between 0 and 15 dB for 18 patients. The coupling quality was higher (worse) than 20 dB in three patients and lower (better) than 0 dB in two patients. The intraoperative VSB-ABR was lower (better) than the actual coupling quality by more than 10 dB in three patients (13%) and higher (worse) than the actual coupling quality by more than 10 dB in two patients (9%). In the remaining 18 patients (78%) the intraoperative VSB-ABR was in line with the coupling quality by less than ± 10 dB. The Bland–Altman analysis of the data (see Fig. 5b) showed a mean difference between the intraoperative VSB-ABR thresholds and the coupling qualities of 1.6 (± 8.4) dB. There was no statistically significant absolute systematic bias, i.e., the mean of the difference was not statistically different from 0 (*t*(22) = 0.905, *p* = 0.375). The limits of agreement were 18.2 and − 15 dB, exceeding the ± 10 dB maximum allowed difference. The Bland–Altman analysis did not reveal a proportional error, i.e., the difference between the intraoperative VSB-ABR and coupling quality was independent of the magnitude of the two measures (the average), the variation was constant.

The outliers exceeding the ± 10 dB tolerance were identified as three patients where the intraoperative VSB-ABR was lower (better) (patients #11, #20, and #22) and two patients where the intraoperative VSB-ABR was higher (worse) (patients #7, and #10) than the coupling quality. The analysis of the postoperatively measured VSB-ABR for these patients revealed that the difference between the postoperative VSB-ABR threshold and the coupling quality reduced to 3.33 and − 1.67 dB for patients #10 and #11 but remained unchanged for patients #20 and #22. For patient #7 the postoperative VSB-ABR was not measured.

## Discussion

The results of the multicenter study show that the introduced method was applicable for measuring intraoperative auditory brainstem responses to stimulation by a Vibrant Soundbridge middle ear implant (intraoperative VSB-ABR) in a series of patients at different centers and with different coupling modalities. Response thresholds could be measured in all except one patient where no responses were obtained (threshold > 30 dB nHL). However, in this patient, the postoperative coupling quality was shown to be insufficient at 36.7 dB nHL postoperatively.

A method for intraoperative quantification coupling quality has not been available before. Verhaegen et al. described a detailed calibration procedure for their set-up but the study did not aim to measure the absolute coupling quality but to conduct a relative assessment [20]. Geiger et al. used a VSB optimized chirp stimulus calibrated according to threshold measurements in implanted patients [24, 25]. They found a significant correlation between VSB-evoked ABR and BC thresholds but not between the ABR and the VIB thresholds. Radeloff et al. used the same experimental set-up as described in this study and recorded CAPs in VSB patients intraoperatively [21]. However, the vibrogram measurement was not available by the time.

The results of this study are the first to show that a quantification of coupling quality was possible. The Bland–Altman analysis of the data—comparing the intraoperative VSB-ABR and the coupling quality, determined by the difference between the vibrogram (VIB) and bone conduction (BC) thresholds, as the gold standard—revealed no statistically significant bias. The mean difference was 1.6 dB and, thus, within measuring accuracy. The VSB-ABR method was independent of the magnitude of the measured thresholds, so that a proportional error could be excluded. However, the limits of agreement exceeded the ± 10 dB maximum allowed difference which was defined as the difference which is not clinically important, indicating that the methods did not agree by an acceptable amount. For further analysis, the five outliers for which the intraoperative VSB-ABR deviated from the coupling quality by more than 10 dB were identified. In two patients (one false positive and one false negative), the postoperative VSB-ABR threshold was in agreement with the coupling quality. In these patients, the VSB-ABR method was feasible to determine the coupling quality but the coupling might have changed before the postoperative testing. However, in the other two patients, the postoperative VSB-ABR did not change compared to the intraoperative VSB-ABR, so that the difference between VSB-ABR and coupling quality of more than ± 10 dB remained unchanged and the discrepancy could not be explained by a change of coupling quality over time.

Comparing the results of this study to the consistency of ABR and behavioral thresholds reported in other studies revealed similar variations between objective and behavioral thresholds. The general feasibility of ABR and consistency with behavioral thresholds has been shown by significant correlations between the thresholds. Cho et al. reported correlation coefficients between 0.43 at 500 Hz and 0.74 at 2000 Hz for CE-chirps and between 0.45 at 1000 Hz and 0.70 at 2000 and 3000 Hz for clicks [29]. The low correlation coefficients indicate wide data spread. Other studies observed that the predictions of behavioral thresholds from ABR thresholds varied with the degree of hearing loss. Gorga et al. found differences between ABR and behavioral thresholds between – 40 and 20 dB with ABR thresholds overestimating behavioral thresholds in cases of normal hearing and underestimating behavioral thresholds in hearing impaired subjects [30]. McCreery et al. reported differences between ABR and behavioral thresholds ranging between − 40 and 20 dB with a mean difference of − 1.2 dB [31]. Thus, the results from these studies show that ABR is a predictor for behavioral thresholds but that discrepancies between ABR and behavioral thresholds can occur so that the methods cannot be used interchangeably as was also shown for the results in our study.

Agreement between the intraoperative VSB-ABR thresholds with the coupling quality is assumed to be due to programming of the audio processor to the patients’ BC thresholds, therefore compensating for the hearing loss, and due to the use of calibrated stimuli with insert earphones. However, the microphone aperture of the audio processor does not correspond to the acoustic coupler (of specified 2 cm^3^ cavity and shape) which is normally used for the calibration of earphones. We, therefore, assume that a coincidental cancelation of calibration errors occured, so that the signal at the FMT was in line with the ABR stimulus intensity in the stimulation system and the VSB-ABR thresholds could be considered as a direct indicator for the magnitude of coupling quality.

Thresholds at 500 Hz were excluded from the data analysis and only the 3PTA was considered. This was due to the broad distribution of coupling quality at 500 Hz as well as the frequency-dependent delay of signal transmission with a maximum at 500 Hz. Due to the delay, the broadband CE-Chirp emerged at the FMT as a click-like stimulus. Cebulla et al. measured the frequency-specific delay for their experimental set-up with a newer version of an audio processor, a Samba Hi, and a wireless streamer for signal transmission [24]. The frequency-dependent delay time function is very similar to the results in this study. Cebulla et al., therefore, developed a VSB-specific stimulus compensating for the delay times to resemble the original CE-Chirp. Despite the general feasibility of the method introduced in this study, the information about the coupling quality at 500 Hz requires alternative stimuli which could be achieved by modifying the VSB-Chirp introduced by Cebulla et al. according to the experimental set-up of this study.

Other technical drawbacks of the set-up were observed in the technical examination and during VSB-ABR measurements. The output saturated for ABR stimulus intensities between 35 and 40 dB nHL. Surprisingly, despite the deactivation of output limitation, the maximum output for programming the AP404 to BC thresholds of 0 dB HL was 15 dB lower than for 20 and 40 dB HL BC settings of the audio processor. However, the saturation between 35 and 40 dB nHL ABR stimulus intensity was not critical for the method itself, since the VSB-ABR threshold was a direct indicator for the magnitude of coupling quality and coupling qualities lower (better) than 20 dB are desirable for sufficient postoperative audiological results with the VSB [15]. VSB-ABR thresholds higher than 30 dB nHL are not intended when applying the method and would—in practice—require repositioning of the FMT. However, potential pitfalls are given by the missing telemetry function of the set-up. This requires optimal positioning of the audio processor over the implant so that signal transmission and auditory stimulation via the implant is provided. Otherwise, this can lead to false negative outcome. False-negative results due to the unlikely event of implant failure cannot be detected with this set-up either. This still requires the use of a QuickCheck device (MED-EL, Innsbruck, Austria). Moreover, the signal amplitude which is dependent on skin flap thickness was not considered in the set-up. A possible solution was described by Ghoncheh et al. with a precision driver device determining the distance between the transmitter coil and the receiver coil in the implant and compensating for transmission loss [32]. They found that the distance can be up to 15 mm during surgery resulting in transmission loss of 11 dB. The authors did not perform measurements in patients and in AMEI but proposed that the method would also be applicable in AMEI.

It was observed that six of 30 patients with intraoperative VSB-ABR measurements had to be excluded from the final analysis due to temporary or permanent deterioration of the postoperative 4PTA BC thresholds by more than 10 dB compared to the preoperative 4PTA BC thresholds. A deterioration by more than 10 dB was chosen as the cut-off criteria, because the applicability of the introduced method, i.e., the calibration approach, required stable BC thresholds. Although the outcome of VSB surgery was not an objective of this study, the high number of patients (20%) with postoperative temporary or permanent threshold deterioration was noticeable. All excluded patients with BC deterioration had undergone RW vibroplasty. The causes for postoperative threshold deteriorations were unknown in five case. No adverse events were reported intraoperatively. In one patient, an unknown OW fistula (after multiple previous surgeries for cholesteatoma) was discovered intraoperatively, leading to postoperative labyrinthitis and complete hearing loss. This patient was treated later with a cochlear implant. In two patients, the BC threshold deterioration was only temporary; in two patients, it was stable afterwards. One patient was lost to follow-up. The audio processors could be successfully fitted in all five patients.

Most studies about the long-term results with the VSB middle ear implant compare mean pre- and postoperative BC thresholds and report no significant differences [16, 19, 33] but do not specify the percentage of patients with BC deterioration. Spiegel et al. reported deterioration of BC thresholds in 11.1% of patients with incus vibroplasty and in 20.0% with RW vibroplasty, which appears in line with our findings [34]. A deviation of 15 dB was considered clinically relevant in their study so that the percentage of patients with BC deterioration by more than 10 dB is potentially higher. In a prospective multicenter study, Zahnert et al. reported postoperative BC deterioration of 20 dB in one patient (3%) with RW vibroplasty [35].

## Conclusions

This is the first study to describe a method which has the potential to quantify the coupling quality of the Vibrant Soundbridge middle ear implant during the surgery. The method was evaluated by collecting data of patients with various coupling modalities and in several centers. Intraoperative auditory brainstem responses were recorded to stimulation by the implanted transducer, driving the implant using a modified audio processor programmed to the patient’s bone conduction thresholds and fitted with insert earphone sound tubes attached to its microphone. The response thresholds recorded in this set-up have been shown to predict the coupling quality. The variation was within the general precision of auditory brainstem responses to predict behavioral thresholds. The method is a tool for the intraoperative assessment of coupling quality and can help the surgeon together with the audiologist to find the optimal position of the transducer or the transducer–coupler assembly. The method is feasible to provide an optimal surgical basis for good postoperative audiological results and, therefore, to improve intraoperative quality control so that revision surgeries due to insufficient coupling can be avoided.

## Data Availability

The datasets generated for this study are available on request to the corresponding author.
